# Synthesis of hydrotalcite-type mixed oxide catalysts from waste steel slag for transesterification of glycerol and dimethyl carbonate

**DOI:** 10.1038/s41598-020-67357-z

**Published:** 2020-06-24

**Authors:** Guanhao Liu, Jingyi Yang, Xinru Xu

**Affiliations:** International Joint Research Center of Green Energy Chemical Engineering, East China University of Science and Technology, Meilong Road 130, Shanghai, 200237 People’s Republic of China

**Keywords:** Environmental sciences, Chemistry, Materials science

## Abstract

The mixed metal oxides S-CaMgAl MO prepared by acidolysis, coprecipitation and calcination under different temperatures from S95 steel slag of Shanghai Baosteel Co., Ltd. were used to catalyze the transesterification of dimethyl carbonate (DMC) and glycerol for synthesizing glycerol carbonate (GC). The catalysts were characterized by EDS, XRD, FT-IR, SEM, CO_2_-TPD and nitrogen adsorption–desorption isotherms. S-CaMgAl MO calcined at 600 °C had excellent catalytic performance due to the large pore size and proper alkalinity. The effects of reaction temperature, reaction time and the amount of catalyst on transesterification were investigated to obtain the optimal reaction conditions. The glycerol carbonate yield reached 96.2% and the glycerol conversion was 98.3% under the condition of 3 wt% catalyst, 1:3 molar ratio of glycerol and DMC, 75 °C reaction temperature and 90 min reaction time. In addition, the GC yield and glycerol conversion still achieved above 90% after five cycles of S-CaMgAl MO.

## Introduction

The glycerol is major by-product glycerol produced in large quantities with the rapid development of biodiesel production which is the focus of global energy and environmental sustainable development as one of the key renewable energy^1^. The efficient conversion of glycerol has become one of the main bottlenecks against the sustainable development of biodiesel industry^2,3^.

Glycerol carbonate (GC) is one of the most promising derivatives of glycerol. glycerol carbonate has been widely used in the field of solvents, elastomers, surfactants, adhesives, inks, paints, lubricants, electrolyzers and personal care products due to its excellent biodegradability, water solubility, low flammability, low viscosity and low toxicity. Furthermore, GC molecules contained hydroxyl group and carbonate group exhibited high reactivity with alcohol, amine, carboxylic acid, ketone, isocyanate and so on^4^, thus the preparation of GC from glycerol presents a remarkable application prospect.

There have been several main methods to prepare GC from glycerol at present including photogasification of glycerol and phosgene^5^, direct carbonylation of glycerol and CO_2_^6^, pyrolysis of urea and glycerol^7^ and transesterification of glycerol with propylene carbonate (PC) or dimethyl carbonate (DMC)^8^. The transesterification of glycerol with DMC over alkaline catalysts is considered a more effective method for the synthesis of GC under mild conditions^9,10^. Pacharaporn^11^ and Sreerangappa^12^ synthesized GC from glycerol and DMC over NaAlO_2_. The GC yield was 85% and 94% under different conditions respectively. The toxic substances, high temperature or other harsh reaction conditions are not needed in this process^13–15^.

Heterogeneous solid base catalysts have shown great potential application in glycerol transesterification because of their easy separation. Using industrial wastes to prepare heterogeneous solid base catalysts is beneficial to environmental protection, resource recycling and sustainable development. Shikhaliyev^16^ prepared porous biochar from fishery waste to catalyze the synthesis of glycerol carbonate. Its effective catalytic component was calcium-based oxide. Okoye^17^ used the calcined blast furnace slag impregnated with NaOH solution to catalyze the esterification of glycerol and dimethyl carbonate.

Steel slag is one of the main solid wastes in the iron and steel industry. Its fundamental components include CaO, SiO_2_, Al_2_O_3_ and MgO. Most steel slag has been used as hydraulic cement, concrete, building materials and so on at present. However, there have been some disadvantages in the application of steel slag such as limited utilization ways and low utilization rate^18^. A large number of unused steel slag has not only occupied valuable land resources, but also caused environmental pollution. The synthesis of novel multi-functional materials from the rich metal resources in steel slag can provide a new idea for the full use of steel slag and greatly reduce the preparation cost of corresponding materials^19^.

Hydrotalcite-like compounds, also known as layered double hydroxides (LDHs), belongs to anionic layered compounds. [M^II^_1-x_M^III^_x_ (OH)_2_]_x+_[A]_x/n_·mH_2_O can be used to describe LDHs. The closer radius of M^II^ and M^III^ in hydrotalcites is generally more favorable to the formation of stable laminate structures^20,21^. LDHs are widely used in environmental remediation, catalysis, pharmaceutical and biological preparation due to the exchangeability of interlayer anions^22–25^. Kuwahara^26^ prepared Ca-Al-Cl hydrotalcite from blast furnace slag and used it to adsorb phosphate in water. Its adsorption capacity was more than three times that of Mg–Al hydrotalcite. The prepared hydrotalcites were also used to trap CO_2_^27^. The mixed oxide prepared by calcination of hydrotalcites was often used as catalysts due to the high basicity. Algoufi^28^ synthesized the Sr-Al mixed oxide and investigated the catalytic effect of the two elements ratio on the esterification. Parameswaram^29^ reported transesterification of glycerol with dimethyl carbonate for the synthesis of glycerol carbonate over Mg-Zr-Sr mixed oxide catalysts. Liu^30^ synthesized glycerol carbonate by transesterification of glycerol with dimethyl carbonate over Mg–Al mixed oxide catalysts.

The materials prepared from steel slag are rich in calcium. The catalyst with high calcium content still has serious stability problems. In this paper, calcium-based catalysts were synthesized by calcining hydrotalcites from steel slag acidolysis to achieve the high-efficient conversion of glycerol as the high value-added by-product of biodiesel production. The catalytic performance and the stability of hydrotalcite-type mixed oxide in the transesterification reaction of glycerol and DMC to prepare GC was investigated.

## Experimental

### Materials

The steel slag used in this study was Type S95 steel slag from Baosteel Co., Ltd. in Shanghai. The raw steel slag was white powder with small particle size. Its composition was analyzed by Energy Dispersive Spectrometry. Table 1 demonstrated that the raw material contained rich metal elements. The mass fraction of CaO, MgO and Al_2_O_3_ was 53.87%, 6.09% and 12.13% respectively. Glycerol (AR), dimethyl carbonate (AR), NaOH (AR), Na_2_CO_3_ (AR) and HNO_3_ (68 wt%) were purchased from Aladdin Reagent Co. Ltd (China).

**Table 1:** Composition of S95 steel slag.

Component	CaO	SiO_2_	Al_2_O_3_	MgO	Fe_2_O_3_	TiO_2_	Others
wt%	53.87	24.52	12.13	6.09	1.14	0.63	1.62

### Catalyst preparation

The hydrotalcites were prepared by coprecipitation method after acidolysis of steel slag and calcined at different temperatures to obtain various mixed oxide catalysts. The metal elements in the steel slag can be extracted into acidolysis solution while SiO_2_ remained in the filter residue due to its insolubility in acidolysis solution. 50.0 g Type S95 steel slag was weighed and added to 400 mL beaker. 200 mL nitric acid solution (7.6 mol/L) was slowly poured into it in order to ensure that the ratio of nitric acid and steel slag was 30 mol/kg. The steel slag was kept acidolysis for 25 min at 25 °C with continuous stirring. The acidolysis solution was obtained by filtration using Buchner funnel.

The hydrotalcites based on steel slag was prepared by coprecipitation. NaOH and Na_2_CO_3_ at the molar ratio of 16:1 were dissolved in deionized water to prepare the alkaline solution. About 50 mL deionized water was added into a 250 mL three-neck flask and remained 70 °C by heating with stirring evenly. The acidolysis solution and the alkali solution were mixed by the double-titration coprecipitation method. The pH value of the solution was kept 10–11 during the titration. The white turbid liquid was stirred for 1 h at 70 °C after titration and then aged in the thermostatic waterbath of 70 °C for 12 h. The steel slag based hydrotalcites S-CaMgAl-CO_3_ LDH can be obtained after filtering, washed and drying in the vacuum oven at 70 °C for 12 h. The steel slag based mixed oxides S-CaMgAl MO were prepared via calcination of hydrotalcite samples in muffle furnace at different temperatures for 6 h.

### Characterization methods

The crystallite sizes and morphologies of solid catalyst were characterized by X-ray diffraction (XRD) on Rigaku D/max2550VB/PC with CuKα radiation (λ = 0.154 nm, 100 mA, 45 kV). The scanning range was 0°–80° (2θ) with a rate of 0.02°/s. Surface elements were measured by Edax Falcon energy dispersive spectrometer (EDS). The pore size distribution and surface area of catalysts were obtained by Brunauer–Emmett–Teller (BET) method on Micromeritrics ASAP-2400. The infrared absorption spectra of the samples were determined by Fourier transform infrared spectroscopy (FT-IR) by KBr pellet. S-3400N scanning electron microscope (SEM) was used to observe the surface structure and shape of samples. The basicity of catalysts was measured by Temperature Programmed Desorption of carbon dioxide (CO_2_-TPD, Micromeritics Autochem 2920). The basic strength of catalysts was determined qualitatively by identifying color change of Hammett indicators including phenolphthalein (H_ = 9.3), 2.4-dinitroaniline (H_ = 15.0), 4-nitroaniline (H_ = 18.4) and aniline (H_ = 27.0).

### Transesterification procedure

Glycerol and dimethyl carbonate were added into the flask. The flask with stirring and reflux device was placed in a constant temperature water bath. A certain amount of catalyst was added to the reaction system after heating to the set temperature. The transesterification was carried out under nitrogen atmosphere. The solid catalyst and the liquid mixture were separated by centrifugation. The product mixture was analyzed by gas chromatograph (Jinghe GC-7860) with flame ionization detector (FID). The type of chromatographic column was HP-PONA (50 m × 0.200 mm × 0.50 μm). The injection temperature and detector temperature were 325 °C and 280 °C respectively^31^. The calculation formulas of Glycerol conversion rate (Eq. 1), GC yield (Eq. 2) and GC selectivity (Eq. 3) were as follows.

1 $$ Glycerol\,conv.\,{(}\% {)} = \frac{{X_{{GLY_{0} }} - X_{{GLY_{i} }} }}{{X_{{GLY_{0} }} }} \times 100\% $$

2 $$ GC \,yield\,{(}\% {)} = \frac{{X_{{GC_{{}} }} }}{{X_{{GLY_{0} }} }} \times 100\% $$

3 $$ GC\, selectivity\,(\% ) = \frac{GC \,yield}{{Glycerol\, conv.}} \times 100\% $$

## Results and discussion

### Effects of calcination temperature on catalyst basicity and transesterification

S-CaMgAl MO prepared by S95 steel slag can be used as solid base catalysts since the steel slag contained a large number of metal elements. The esterification reaction of dimethyl carbonate and glycerol was catalyzed by S-CaMgAl MO calcined at 100 °C 350 °C, 600 °C and 850 °C respectively when the molar ratio of DMC and glycerol was 3:1, the catalyst dosage was 3 wt%, the reaction temperature was 75 °C and the reaction time was 90 min. The results were shown in Table 2. The basic strength and amount of S-CaMgAl MO were also measured in order to analyze the relationship between the catalytic effect and the basicity of catalysts. The basic amount of S-CaMgAl MO calcined at 100 °C, 350 °C, 600 °C and 850 °C was 0.87 mmol/g, 0.94 mmol/g, 0.99 mmol/g and 1.07 mmol/g respectively. The catalyst basicity and the conversion of glycerol increased with the increase of calcination temperature. However, the yield of glycerol carbonate decreased from 96.2 to 93.3% and TON reduced from 351 to 317 when the calcination temperature of catalysts changed from 600 to 850 °C due to the further formation of glycidol in the strong alkaline environment. The enhanced basicity was favorable to the conjugation of carboxylic acid ions. The gained stability of COO^−^ led to the decarboxylation of glycerol carbonate.

**Table 2:** Effect of different calcination temperature on glycerol conversation and GC yield.

Catalyst (calcination temperature)	S-CaMgAl MO (100 °C)	S-CaMgAl MO (350 °C)	S-CaMgAl MO (600 °C)	S-CaMgAl MO (850 °C)
Glycerol conv. (%)	81.6	92.5	98.3	98.4
GC yield (%)	80.9	91.3	96.2	93.3
GC selectivity	99.1	98.7	97.9	94.8
TON	55	107	351	317
Basic strength (H_)	H_ < 9.3	9.3 < H_ < 15.0	15.0 < H_ < 18.4	18.4 < H_ < 26.5
Basic amount (mmol/g)	0.87	0.94	0.99	1.07

The metal cations were filled in the appropriate gaps of O^2−^ densely packed according to their ion radius to form the special structures of mixed oxides, which was obviously different from the mixture of metal oxides. The preparation of mixed oxides was related to the calcination temperature closely. 600 °C was the optimal temperature for the catalyst synthesis in this experiment and its catalytic performance was also the best. Therefore, S-CaMgAl MO calcined at 600 °C was chosen for further experiments.

### Catalyst characterization

#### XRD analysis

**Figure 1:**
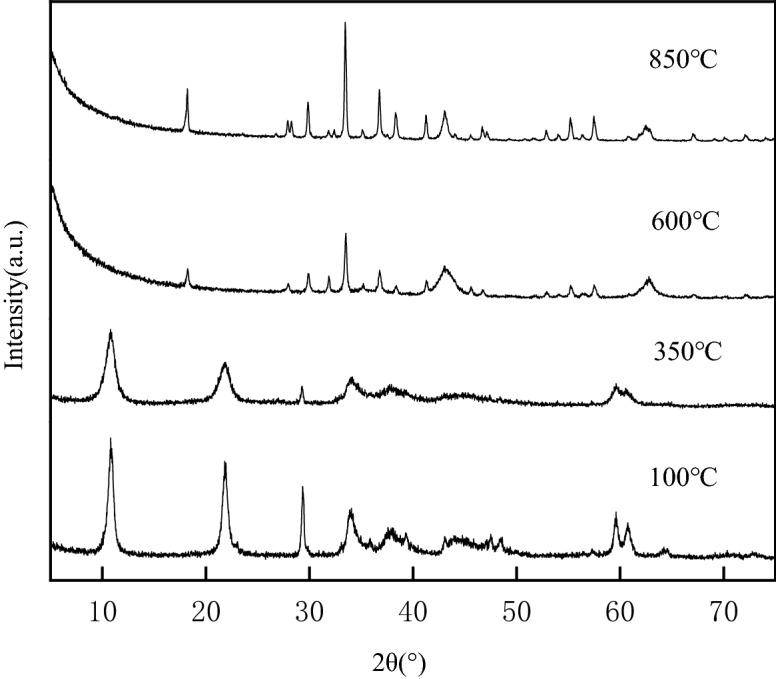
XRD patterns of S-CaMgAl MO.

Figure 1 showed the X-ray diffraction patterns of S-CaMgAl MO samples calcined at different temperatures. The hydrotalcites characteristic symmetrical reflection of (003), (006), (009), (015), (018), (110) and (113) surfaces was observed in the XRD patterns of the samples calcined at 100 °C. The characteristic peaks where 2θ was 29.8°, 33.4° and 46.7° can be attributed to the tetragonal phase of CaO. The peaks of MgO cubic phase were at 43.1° and 62.8°. The crystal surface reflection at 35.1° and 47.1° were related to γ-Al_2_O_3_ phase. The structure and composition of catalysts changed significantly with the increase of calcination temperature. The characteristic peaks of hydrotalcites were weakened while the diffraction peaks of CaO, MgO and Al_2_O_3_ appeared and became strong gradually in the catalyst calcined at 350 °C. The symmetrical reflection of hydrotalcites in the XRD pattern of samples disappeared completely and the catalyst turned into the mixed oxides of CaO, MgO and Al_2_O_3_ when the calcination temperature was 600 °C and 850 °C.

#### FT-IR analysis

**Figure 2:**
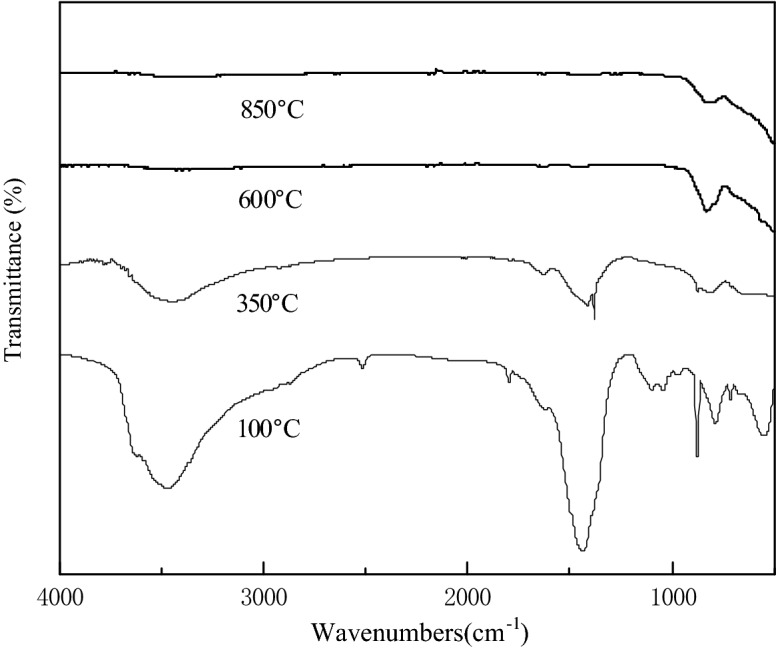
FT-IR spectra of S-CaMgAl MO.

The FT-IR spectra of S-CaMgAl MO samples calcined at different temperatures were displayed in Fig. 2. The stretching vibration peaks near 700 cm^−1^ were caused by Ca-O, Mg-O and Al-O. The peaks observed at low wavenumbers (between 600 and 400 cm^−1^) were corresponded to metal hydroxides Ca(OH)_2_, Mg(OH)_2_ and Al(OH)_3_ when the calcination temperature was 100 °C. The characteristic peak of carbonates was at 1,440 cm^−1^. The absorption peak at 3,470 cm^−1^ was ascribed to O–H group. The stretching vibration peaks of hydroxyl and carbonate ions decreased gradually in the spectra of catalyst calcined at 350 °C due to the removal of crystal water and CO_3_^2−^ in the layers of hydrotalcites by calcination. The absorption peaks of hydroxyl and carbonate ions disappeared completely when the calcination temperature was 600 °C and 850 °C, which indicated that the hydrotalcite material was completely transformed into mixed metal oxides at this point.

#### CO_2_-TPD analysis

**Figure 3:**
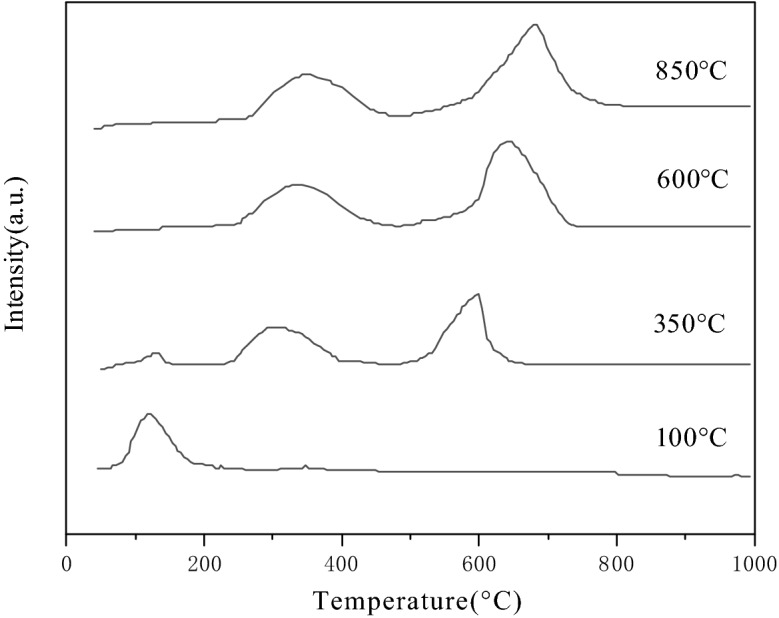
CO_2_-TPD profiles of S-CaMgAl MO.

**Figure 4:**
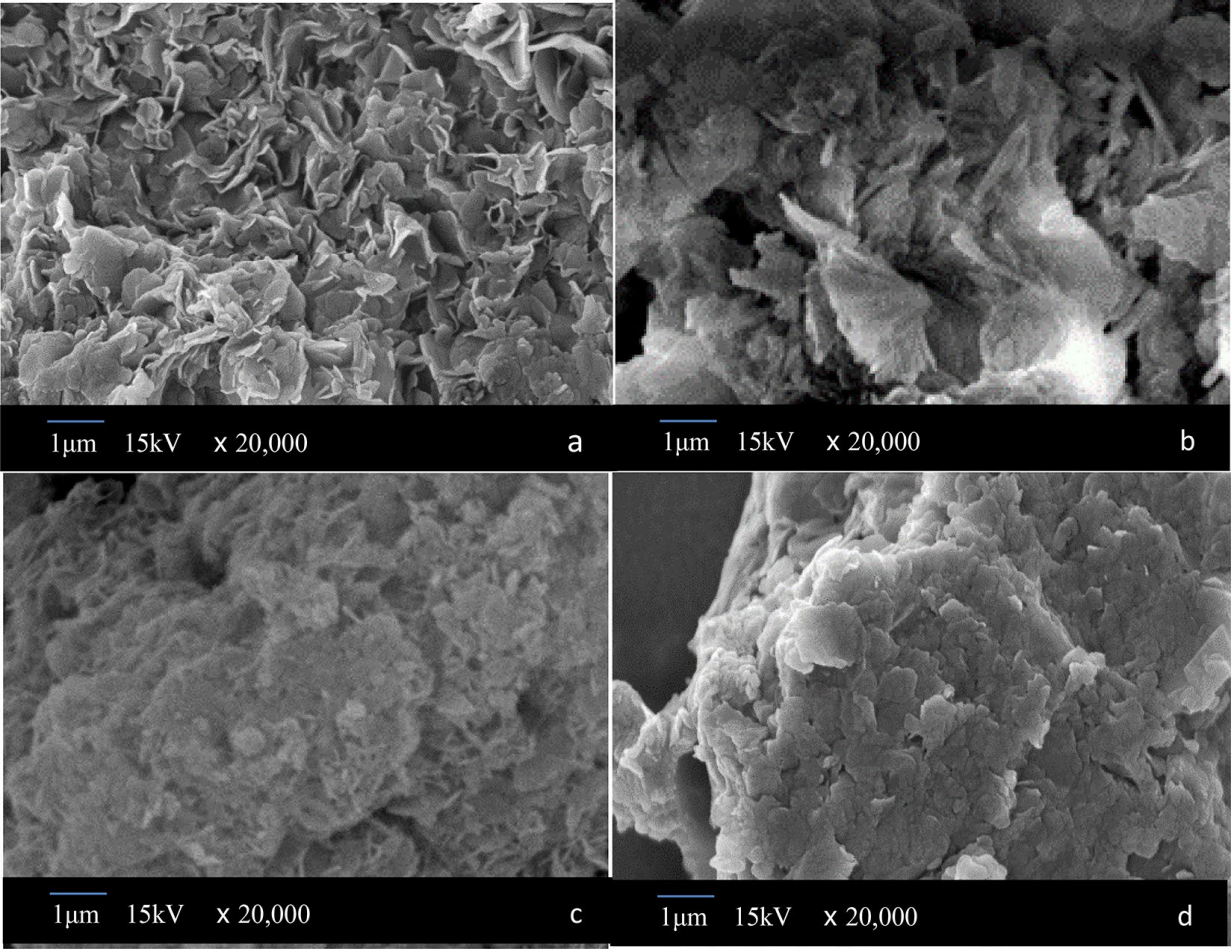
SEM images of S-CaMgAl-CO_3_ LDH (**a**) and S-CaMgAl MO calcined at 350 °C (**b**), 600 °C (**c**) and 850 °C (**d**).

Figure 4 showed the CO_2_-TPD profile of S-CaMgAl MO samples. Generally, the desorption peak of CO_2_ at lower than 200 °C, 300 °C to 450 °C and higher than 450 °C match weak, moderate and strong basic sites respectively^32^. The CO_2_ desorption peak near 200 °C was corresponded to the weak alkaline center OH^−^ on the catalyst surface. The strength of this peak became weakened gradually and disappeared with the rise of calcination temperature. The desorption of CO_2_ at about 400 °C was attributed to the metal oxygen (M–O) bond. This peak was broad since there were several metal oxides in the catalysts. The CO_2_ desorption peak around 600 °C was ascribed to the strong alkaline Center O^2−^. The desorption peak shifted towards high temperature gradually with the increase of calcination temperature in Fig. 3, indicating that the basicity of S-CaMgAl MO was also enhanced.

Metallicity is the ability of releasing electrons for an atom in chemical reactions. The strong metallicity means the strong electron-loss ability and the strong alkalinity of corresponding alkali. The metallicity of Ca is stronger than magnesium, thus the alkalinity of mixed oxides can be improved by partially substituting calcium for magnesium.

The formation of O^2−^ can be attributed to the special structures of hydrotalcites and the mixed oxides. The metal elements and oxygen elements were closely packed in the oxide lattices of general oxides regularly. However, part of Mg^2+^ were replaced by Al^3+^ in the preparation process of mixed oxides. In other words, the original space of three Mg^2+^ was occupied by two Al^3+^ in the oxide frame in order to achieve the charge conservation. The lattice disorder due to the dislocation of cations resulted in serious Schottky Defects. Mg^2+^ on the catalyst surface moved inside to fill the inevitably generated cation vacancy of in lattices under the thermal movement and Coulomb force, which led to the partial unsaturated adjacent oxygen elements on the surface and the strong alkaline O^2−^ further^33^.

#### Element analysis

The surface elements of S-CaMgAl-CO_3_ LDH and S-CaMgAl MO were analyzed in Table 3. The mass fraction of Ca, Mg, Al and O in S-CaMgAl-CO_3_ LDH was 39.38%, 11.86%, 13.51% and 32.61% respectively. The molar ratio of divalent metal and trivalent metal was between 2 and 4, indicating that the prepared S-CaMgAl-CO_3_ LDH was stable^34,35^. Moreover, the proportion of divalent metal ions and trivalent metal ions on S-CaMgAl MO was similar to S-CaMgAl-CO_3_ LDH, which meant there was little loss of metal elements in the synthesis of the mixed oxides and the obtained catalyst met the expectation.

**Table 3:** Element composition of S-CaMgAl-CO_3_ LDH and S-CaMgAl MO.

Element wt%	Ca	O	Al	Mg	Ca^2+^:Mg^2+^:Al^3+^ (molar ratio)	M^2+^/M^3+^
S-CaMgAl-CO_3_ LDH	39.38	32.61	13.51	11.86	1.97:0.98:1	2.95
S-CaMgAl MO	41.57	25.43	14.36	12.50	1.95:0.98:1	2.93

#### SEM analysis

The morphology of hydrotalcite and mixed oxide calcined at different temperatures were analyzed by Scanning Electronic Microscopy (SEM). As shown in Fig. 4, The hydrotalcite sample S-CaMgAl-CO_3_ LDH displayed obvious characteristics of hollow "petals" layered structures. The layered structure of hydrotalcites disappeared gradually after calcination and the surface of corresponding mixed oxide S-CaMgAl MO presented abundant pore structures.

#### N_2_ adsorption–desorption isotherms analysis

**Figure 5:**
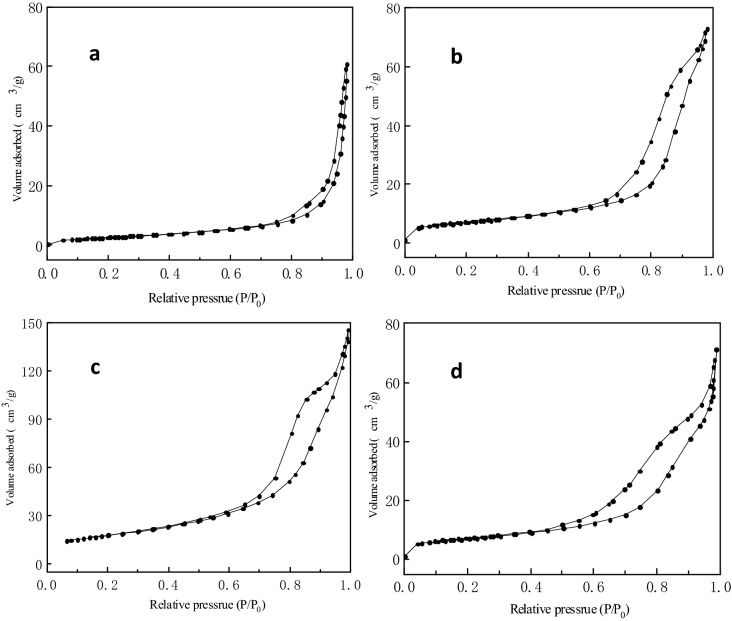
N_2_ adsorption–desorption isotherms of S-CaMgAl-CO_3_ LDH (**a**) and S-CaMgAl MO calcined at 350 °C (**b**), 600 °C (**c**) and 850 °C (**d**).

The structure parameters of catalyst samples with were analyzed by N_2_ adsorption–desorption isotherms. The results were shown in Table 4 and Fig. 5. The specific surface area of S-CaMgAl MO decreased due to the destruction of original layered structures in the hydrotalcite material S-CaMgAl-CO_3_ LDH and the removal of OH^−^ and CO_3_^2−^ anions in the interlayer during the calcination process. However, the mesoporous mixed oxide samples presented larger pore size than hydrotalcites. In addition, S-CaMgAl-CO_3_ LDH exhibited a Type IV isotherm with H3 hysteresis loop due to the typical layered structures of hydrotalcites while the N_2_ adsorption–desorption isotherms of S-CaMgAl MO belonged to Type IV isotherm with H2 hysteresis loop which was characteristic isotherm of mesoporous materials with irregular pore structure^36^.

**Table 4:** Textural properities of S-CaMgAl-CO_3_ LDH and S-CaMgAl MO.

Calcination temperature (°C)	BET surface area (m^2^/g)	Average pore size (nm)
100	102.98	13.19
350	73.56	20.33
600	40.27	31.28
850	10.74	33.68

### Effects of reaction conditions on transesterification between DMC and glycerol

In the transesterification of glycerol and DMC, the basic active sites especially the highly alkaline O^2−^ and Ca–O in the mixed metal oxides split the hydrogen and oxygen of hydroxyl on the glycerol molecules to form the nucleophilic intermediate alkoxy. Then the nucleophilic alkoxy group attacked the carbonyl carbon of DMC thus resulted in the formation of hydroxyalkyl carbonate and methoxy anion. Further molecular reactions generated the target product GC and two methanol molecules as by-products. Finally, GC was separated from the metal sites of the catalyst then the catalyst was used in the next reaction cycle. The optimal reaction conditions were investigated in order to achieve the highest GC yield in this section.

#### Effects of catalyst dosage on glycerol conversion and GC yield

**Figure 6:**
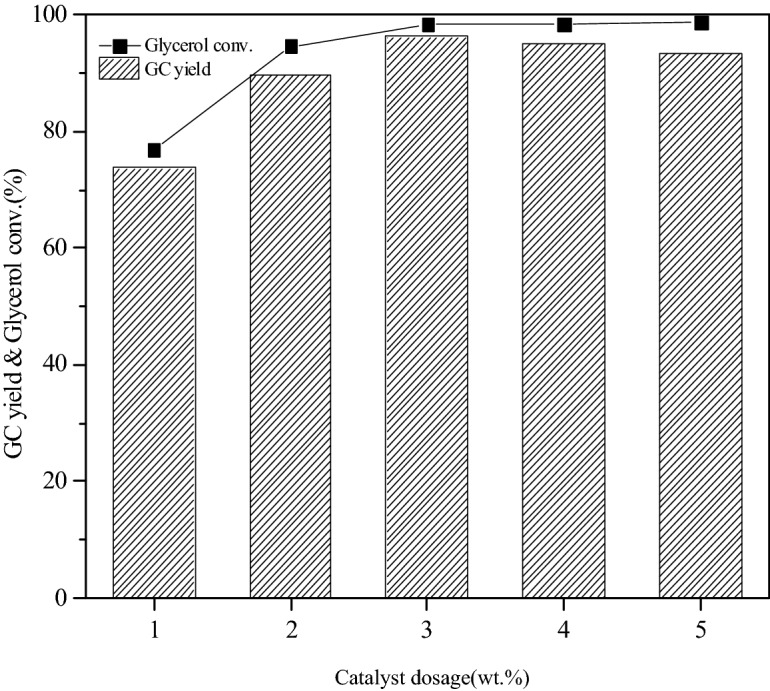
Effect of catalyst dosage on glycerol conversation and GC yield.

The influence of catalyst dosage on the transesterification was investigation under the conditions that the molar ratio of DMC and glycerol was 3:1, the reaction temperature was 75 °C and the reaction time was 90 min. The change of glycerol conversion and GC yield with the amount of catalyst was shown in Fig. 6. The number of basic active sites in the reaction system which was necessary for the glycerol deprotonation and subsequent reaction with DMC to produce glycerol carbonate increased with the increase of catalyst dosage. Thus the conversion of glycerol varied from 76.9 to 98.3% and the GC yield increased from 73.7 to 96.2% when the catalyst dosage rose from 1 to 3%. The conversion of glycerol increased not obviously when the catalyst dosage mounted up continuously because the particle agglomeration caused by excessive catalyst hindered the mass transfer from catalyst body to the active site and prevented the further conversion of glycerol. Moreover, the extra catalytic active sites provided places for the generation of glycidol via decarbonylation of GC, thus the yield of GC was reduced.

#### Effects of reactant molar ratio on glycerol conversion and GC yield

**Figure 7:**
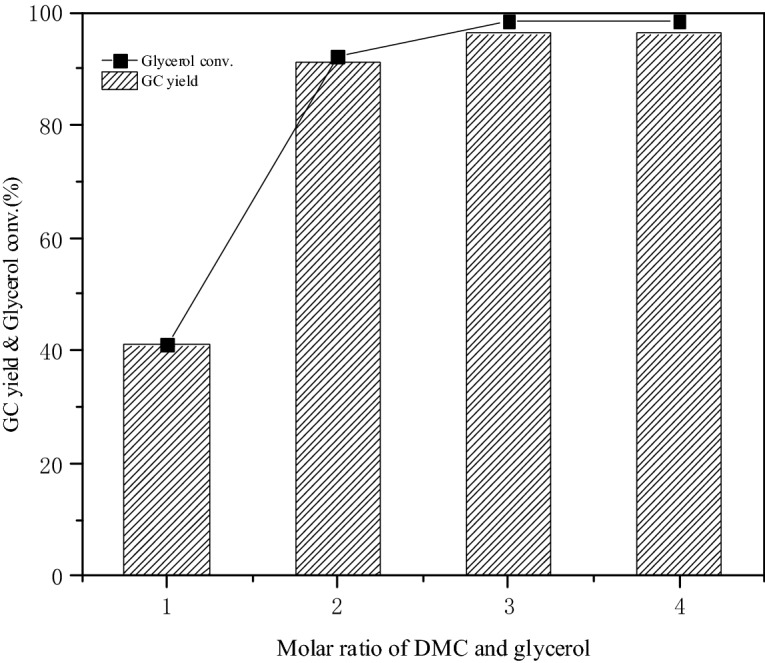
Effect of reactant molar ratio on glycerol conversation and GC yield.

Figure 7 showed the effect of DMC-to-glycerol molar ratio on glycerol conversion and GC yield when the catalyst dosage, reaction temperature and reaction time was constant. The conversion rate of glycerol and the yield of GC are both 41.0% at 1:1 molar ratio of DMC to glycerol. The excessive DMC helped the chemical equilibrium shift to the direction of GC synthesis since the transesterification of glycerol and DMC was reversible. The conversion of glycerol and the yield of GC were 98.3% and 96.2% respectively when the mole ratio of DMC and glycerol rised to 3:1. However, there was no significant change in the glycerol conversion rate and the GC yield when the molar ratio of DMC and glycerol continued to mounted up. Thus the optimal molar ratio of DMC and glycerol was 3:1 considering the conversion of glycerol, the yield of GC and the cost of the reaction.

#### Effects of reaction temperature on glycerol conversion and GC yield

**Figure 8:**
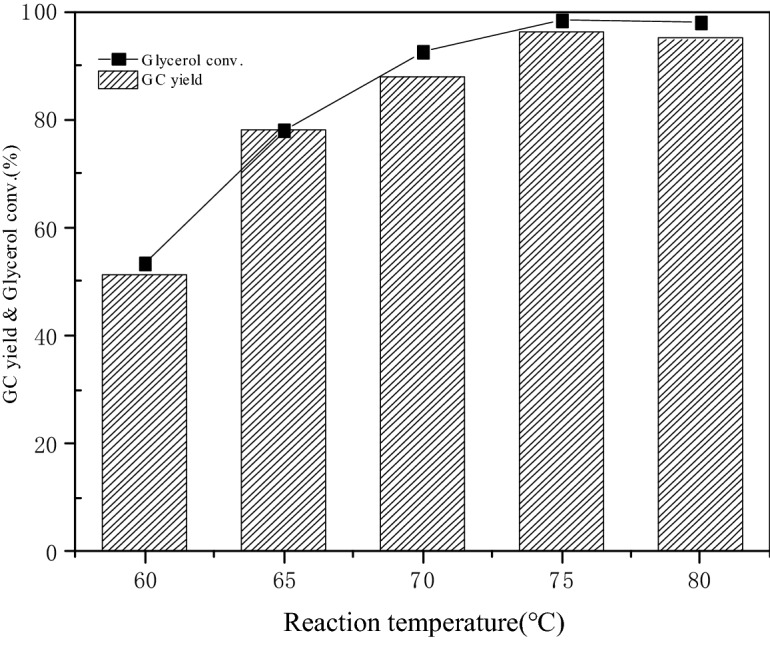
Effect of reaction temperature on glycerol conversation and GC yield.

The effect of reaction temperature ranging from 60 to 80 °C on glycerol conversion and GC yield was displayed in Fig. 8 when the molar ratio of DMC and glycerol was 3:1, the catalyst dosage was 3 wt% and the reaction time was 90 min. The conversion of glycerol increased from 53.3 to 98.3% and the yield of GC rose from 51.4 to 96.2% with the change of reaction temperature from 60 to 75 °C. The molecular velocity and the number of effective collisions between reactant and catalyst increased with the increase of reaction temperature. Thus the glycerol conversion and GC yield increased. There was no significant change in glycerol conversion and GC yield when the reaction temperature continued to rise above 75 °C because the transesterification reached equilibrium basically around this temperature. The viscosity of reactants was no longer the main factor that hindered the shift of chemical equilibrium to the direction of GC synthesis when the reaction temperature was above 75 °C^37^. Meanwhile, side reactions such as the decarbonylation of GC occurred with the rise of reaction temperature. Therefore, 75 °C was chosen as the optimal reaction temperature.

#### Effects of reaction time on glycerol conversion and GC yield

**Figure 9:**
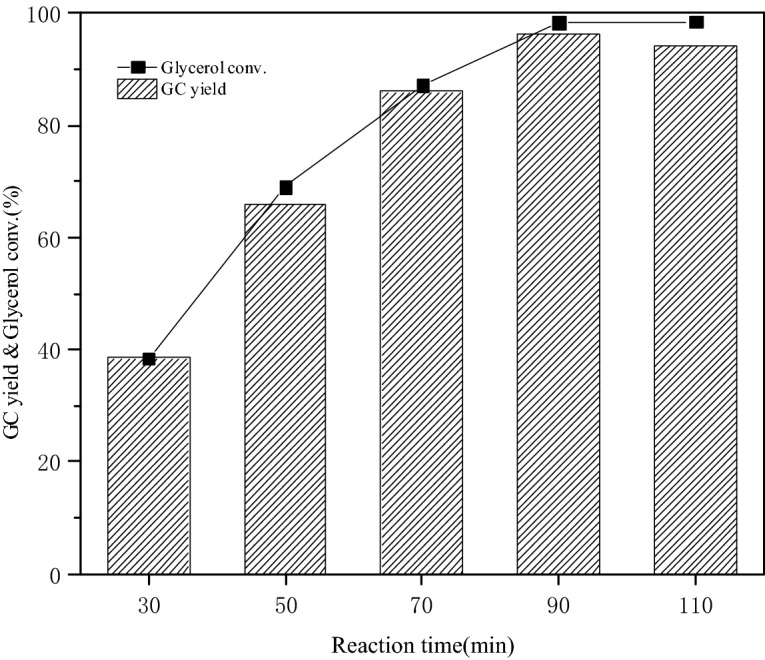
Effect of reaction time on glycerol conversation and GC yield.

Figure 9 presented the influence of reaction time on glycerol conversion and GC yield with 3 wt% catalyst dosage under 75 °C at 3:1 molar ratio of DMC and glycerol. The conversion rate of glycerol and the yield of GC were both at a low level at 30 min. The glycerol was completely converted to GC during this period. The glycerol conversion and GC yield increased significantly from 30 to 90 min. The maximum glycerol conversion rate and GC yield were 98.3% and 96.2% respectively at 90 min. The conversion of glycerol increased while the GC yield decreased slightly when the reaction time mounted up continuously. The optimal reaction condition for the transesterification of glycerol and DMC catalyzed by mixed oxide based on steel slag was 75 °C, 90 min and 3 wt% catalyst dosage from the above experimental results. The catalytic performance of different catalysts was listed in Table 5.

**Table 5:** Comparison of S-CaMgAl MO with reported catalyst in transesterification of glycerol and DMC.

Catalyst	Catalyst dosage (wt%)	Reaction temperature (°C)	Molar ratio of DMC to glycerol	Reaction time (min)	GC yield (%)	Ref
CaO/Al_2_O_3_	9	80	3:1	120	90.6	^9^
Na_2_SiO_3_-200	5	75	4:1	150	95.5	^10^
CaO	3	75	2:1	30	90.2	^13^
NaY zeolite	10	70	3:1	240	80.0	^38^
Calcined dolomite	6	75	3:1	90	94.0	^39^
Mg–Al hydrotalcites	2	100	5:1	540	98.0	^40^
S-CaMgAl MO	3	75	3:1	90	96.2	This work

### Reusability and stability test of S-CaMgAl MO

High stability of catalyst materials was required for the continuous reaction of industrial application to resist leaching of metal active sites and rapid deactivation. Generally speaking, The decrease of activity of solid base catalyst in transesterification of glycerol with DMC can be attributed to the loss of active site in the catalyst or the attachment of organics obstructing the active center to the catalyst surface.

The product mixture obtained from the catalytic reaction was analyzed by ICP-OES to investigated the leaching of metal ions in S-CaMgAl MO. Meanwhile, CaO was used to catalyze the transesterification under the same conditions for comparison. The experimental results showed that when the catalyst was CaO, the concentration of calcium ion in the product mixture was 4.6 mg/L while no metal ion was detected when the reaction was catalyzed by S-CaMgAl MO in Table 6.

**Table 6:** ICP-OES analysis of the product mixture.

Catalyst	Concentration of leaching metal elements (ppm)		
	Ca	Mg	Al
CaO	4.6	–	–
S-Ca/Mg/A1 MO	N.D	N.D	N.D

**Figure 10:**
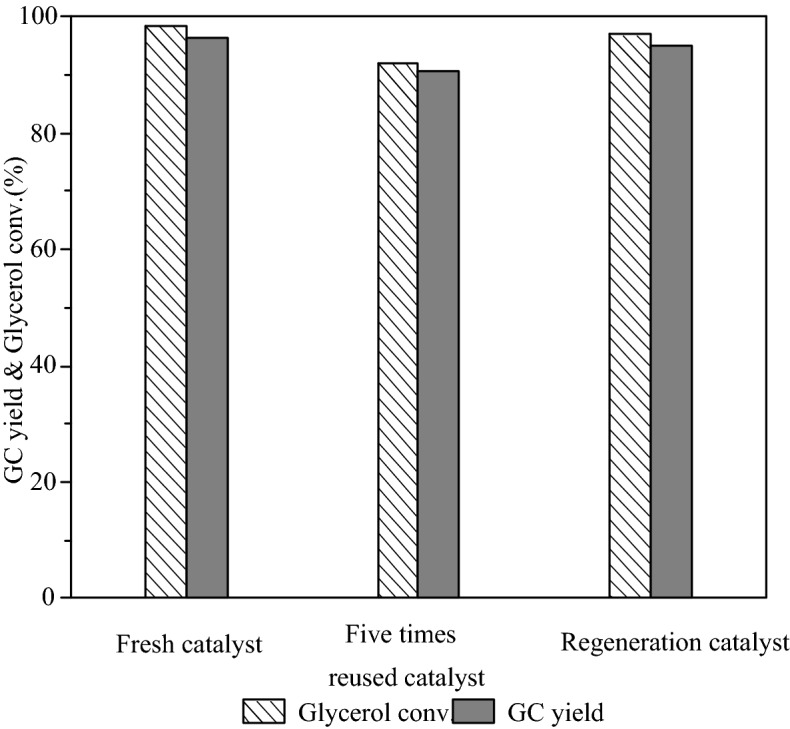
Reusability and regenerability of S-CaMgAl MO.

The reusability and regeneration experiment of S-CaMgAl MO was carried out under the condition of 3 wt% catalyst dosage, 3:1 molar ratio of DMC to glycerol, 75 °C reaction temperature and 90 min reaction time. The catalyst was washed with methanol and dried after each reaction cycle. The five times reused catalyst was calcined at 600 °C for 6 h and then used in the transesterification to test the regenerability of S-CaMgAl MO. Figure 10 showed that S-CaMgAl MO remained 91.9% glycerol conversion and 90.5% GC yield in the fifth cycle. The regeneration catalyst retained 96.9% glycerol conversion and 95.0% GC yield. All indicated excellent reused and regeneration performance of S-CaMgAl MO.

**Figure 11:**
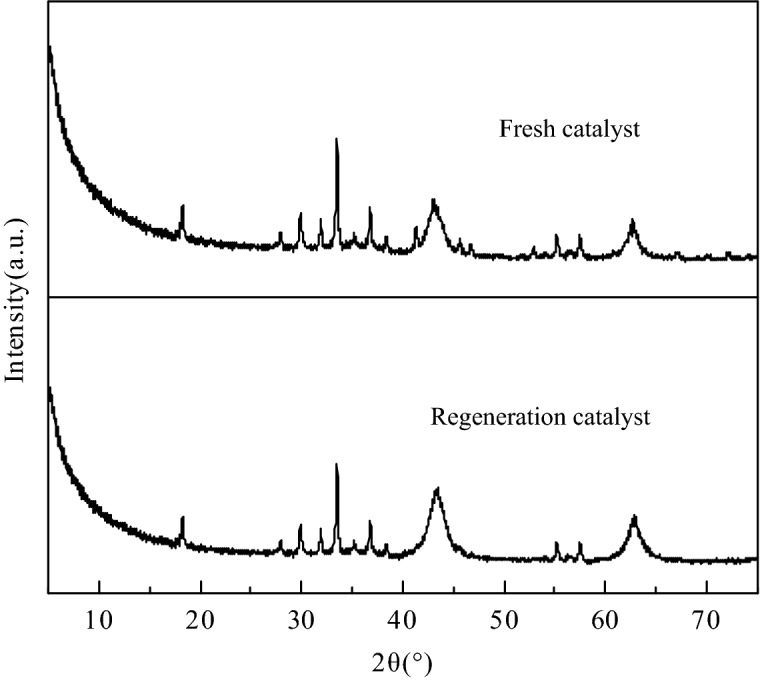
X-ray diffraction patterns of S-CaMgAl MO before and after five times reuse.

**Figure 12:**
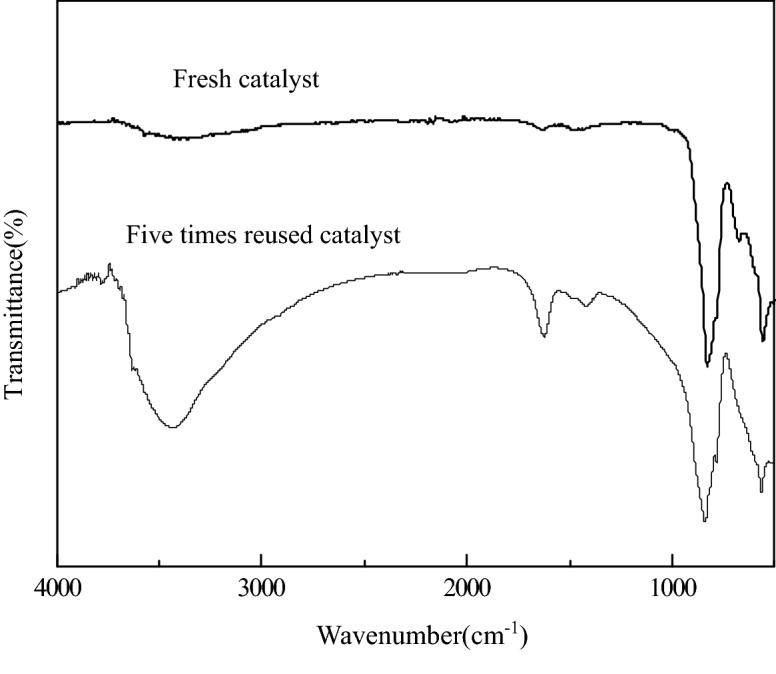
FT-IR spectra of S-CaMgAl MO before and after 5 times reuse.

The XRD patterns of regeneration catalyst and fresh catalyst were contrasted as shown in Fig. 11 to investigate the stability of the catalyst. The results demonstrated that the strength of characteristic peaks corresponded to CaO, MgO and Al_2_O_3_ in the XRD pattern of regeneration catalyst was not weakened significantly compared with the fresh catalyst, which proved that the catalyst had high resistance to metal ion leaching in this reaction system. The strong stability of the catalyst was attributed to the intact microcrystalline structure derived from S-CaMgAl-CO_3_ LDH. The FT-IR spectra of fresh catalyst and five times reused catalyst were also compared in Fig. 12. The wide absorption bands near 3,500 cm^−1^ and 1,500 cm^−1^ caused by hydroxyl and ester group were observed in the spectra of five times reused catalyst, which confirmed the adhesion of organics on the surface and pores of five times reused catalyst.

The surface area of catalyst before and after use was displayed in Table 7. The adhesion of organics resulted in the decreased specific surface area and the worse catalytic activity of S-CaMgAl MO. However, the specific surface area of regeneration catalyst changed little from that of fresh catalyst and the catalytic performance became better after calcination. Hence the deactivated catalyst caused by organics attachment can be restored via calcining at a suitable temperature.

**Table 7:** Surface area of S-CaMgAl MO under different treatment conditions.

Catalyst	BET surface area (m^2^/g)
Fresh catalyst	40.27
Five times reused catalyst	30.16
Regeneration catalyst	38.96

The surface elements analysis of S-CaMgAl MO under different treatment conditions was analyzed. Table 8 showed the carbon element of the catalyst increased by about 5.44 wt% and the Ca element decreased by about 5.50 wt% after five times reuse compared with the fresh catalyst, while there was no striking difference of element contents between the regeneration catalyst and the fresh catalyst. The results indicated that the fall of calcium content was ascribed to the organics adhered to the surface of catalyst after repeated experiments but not the leaching of active sites.

**Table 8:** Element analysis of catalyst samples.

Catalyst	C (%)	O (%)	Ca (%)	Mg (%)	Al (%)
Fresh catalyst	4.18	25.43	41.57	12.50	14.36
Five times reused catalyst	9.62	29.71	36.07	10.94	12.52
Regeneration catalyst	5.07	25.80	40.51	12.00	14.23

CaO with fine catalytic activity would generate little active Ca(OH)_2_ and inactive CaCO_3_ in case of contacting with air. Pure CaO would also be destroyed and converted to ionic state if there was water and acid in the reaction system, which not only reduced the catalyst activity, but also increased the difficulty of catalyst recovery. The novel catalyst combined CaO with MgO and Al_2_O_3_ was prepared in order to prevent the loss of active sites in this paper. The calcium-based catalysts would get deteriorated inevitably after prolonged exposure to the air because of the reaction with water vapor and carbon dioxide. However, the mixed oxide after absorbing certain water or carbon dioxide can be calcined under a suitable temperature to restore the original crystal structures, which was also known as the memory effect of hydrotalcites. Hence the mixed oxide was considered to have resistance to water or carbon dioxide.The composite of different metal oxides existed catalytic synergy and supporting effects, enhancing the anti-leaching ability of active sites and catalytic ability of S-CaMgAl MO.

## Conclusions

Ca-Mg-Al mixed metal oxide catalyst based on steel slag was synthesized at different calcination temperature and used in the transesterification of glycerol and DMC to prepare GC in this paper. S-CaMgAl MO calcined at 600 °C had the best catalytic effects and exhibited high reactivity due to the presence of alkaline earth metal elements. The results showed that the highest yield of GC reached 96.2% and the conversion of glycerol was 98.3% under the condition of 3 wt% catalyst, 3:1 molar ratio of DMC to glycerol, 75 °C reaction temperature and 90 min reaction time. Meanwhile, the crystal structure derived from S-CaMgAl-CO_3_ LDH enhanced the anti-leaching ability of active sites in the catalyst. S-CaMgAl MO achieved 91.9% glycerol conversion and 90.5% GC yield in the fifth reaction cycles in spite of inevitable deactivation. The conversion rate of glycerol and GC yield still reached 96.9% and 95.0% respectively using the regeneration catalyst. Therefore, S-CaMgAl MO has significant industrial application potential in GC synthesis due to the high catalytic activity, good stability, mild reaction conditions and low production cost.
